# Sensitivity to Thyroid Hormone Indices Are Closely Associated With NAFLD

**DOI:** 10.3389/fendo.2021.766419

**Published:** 2021-11-05

**Authors:** Shuiqing Lai, Jiarong Li, Zixiao Wang, Wei Wang, Haixia Guan

**Affiliations:** ^1^ Department of Endocrinology, Guangdong Provincial People's Hospital, Guangdong Academy of Medical Sciences, Guangzhou, China; ^2^ Department of Endocrinology and Metabolism, The First Hospital of China Medical University, Shenyang, China; ^3^ Department of Endocrinology and Metabolism, The First People's Hospital of Ziyang, Ziyang, China; ^4^ Department of Physical Examination Center, The First Hospital of China Medical University, Shenyang, China

**Keywords:** thyroid function, sensitivity to thyroid hormone indices, thyroid feedback quantile-based index, dyslipidemia, non‐alcoholic fatty liver disease

## Abstract

**Background:**

Previous studies on the association between thyroid function and non‐alcoholic fatty liver disease (NAFLD) have contradicted. Acquired resistance to thyroid hormone theory might provide a reasonable explanation for these contradictions. We aimed to analyze the association between sensitivity to thyroid hormone indices with NAFLD.

**Methods:**

A total of 4,610 individuals from the health medical center of the First Hospital of China Medical University were included in this study. The previously used thyroid feedback quantile-based index (TFQI_FT4_) was calculated. Also, we substituted free triiodothyronine (FT_3_) into the TFQI formulas to get the TFQI_FT3_ index. NAFLD was defined using abdominal ultrasound.

**Results:**

Study results showed that FT_3_/FT_4_ and TFQI_FT3_ were positively correlated with the triglyceride (TG), total cholesterol (TC), and low-density lipoprotein cholesterol (LDL-C) levels (*P*<0.05) and negatively correlated with high-density lipoprotein cholesterol (HDL-C) level (*P*<0.05). In contrast, TFQI_FT4_ was positively correlated with HDL-C level (*P* < 0.05). After adjustment for multiple confounders, FT_3_, FT_3_/FT_4_, and TFQI_FT3_ were positively associated with the risks of dyslipidemia and NAFLD (*P* < 0.05). TFQI_FT3_ and FT_3_/FT_4_ performed better than TFQI_FT4_ on ROC analyses for NAFLD prediction, although the diagnostic sensitivity and specificity at the optimal cut-points were low. However, no association was observed between TFQI_FT4_ with the risks of dyslipidemia and NAFLD.

**Conclusion:**

TFQI_FT3_ and FT_3_/FT_4_ can be used as new indicators for predicting dyslipidemia and NAFLD, although with low sensitivity and specificity at the optimal cut-points, while TFQI_FT4_ has insufficient evidence in predicting dyslipidemia and NAFLD.

## Introduction

Non‐alcoholic fatty liver disease (NAFLD) includes a broad range of conditions from fat accumulation within the liver (simple steatosis), liver inflammation (non‐alcoholic steatohepatitis, NASH) through to liver fibrosis and cirrhosis, the latter having an increased risk for progression to hepatocellular carcinoma. What is more, emerging evidence has shown that NAFLD is related to extrahepatic complications such as obesity, type 2 diabetes, cardiovascular diseases, kidney diseases, malignancy, and all-cause mortality (1). Despite this alarming evidence, the nomenclature and the definition of NALFD have not been updated to reflect the latest knowledge. The heterogeneity of the population with NAFLD concerning its causal factors and the comorbidities represents an essential impediment to discovering highly effective medications. Thus, to more accurately reflect the heterogeneity of the disease, the international consensus panel has recently advised using metabolic associated with fatty liver disease (MAFLD) instead of NAFLD (2). Nevertheless, for the sake of this study, we will continue the use of NAFLD, which has been used in our previous data and has been widely accepted in the literature.

The liver plays an essential role in lipid metabolism, including the synthesis and transportation of cholesterol and triglycerides (3). Disorder of hepatic lipid metabolism may precipitate the fat retention within the liver and subsequent development of dyslipidemia and NAFLD. Thyroid function is one of the most important factors regulating liver lipid metabolism. Epidemiological data showed that the prevalence of NAFLD was 27.4–33.1% in the population with euthyroidism, 35.7–36.3% in the population with hypothyroidism, and 11.95-21.5% in the population with hyperthyroidism (4–6). Several studies also demonstrated that free thyroxine (FT_4_) and free triiodothyronine (FT_3_) serum levels were negatively associated with the risk of NAFLD and thyroid-stimulating hormone (TSH) serum levels were positively associated with the risk of NAFLD in the population with thyroid dysfunction (7–11). Furthermore, systematic reviews confirmed the positive association between hypothyroidism and NAFLD risk (12, 13). On the other side, thyroid dysfunctions both in the form of overt and subclinical hypothyroidism were more common among patients with NAFLD (14–16).

However, results from euthyroid patients were inconsistent. From a large cohort study, higher-normal serum FT_3_ and lower-normal serum TSH levels were independently related to a higher incidence of NAFLD (17). Thus, a mild acquired resistance to thyroid hormone might exist in the euthyroid population with NAFLD. So far, however, there has been little research on the association between sensitivity to thyroid hormone indices with the risk of NAFLD. Thyroid Feedback Quantile-based Index (TFQI) was proposed by Laclaustra, a novel index of central sensitivity to thyroid hormone. Laclaustra found that TFQI was related to cardiometabolic health characteristics in the general population (18). Therefore, this cross-sectional study aimed to investigate the direct association of central sensitivity to thyroid hormone (evaluated by TFQI) and peripheral sensitivity to thyroid hormone (evaluated by FT_3_/FT_4_) with dyslipidemia and NAFLD, trying to overcome current contradictions about the association between circulating thyroid hormone levels and hepatic alterations.

## Methods

### Subjects and Study Design

**Figure 1:**
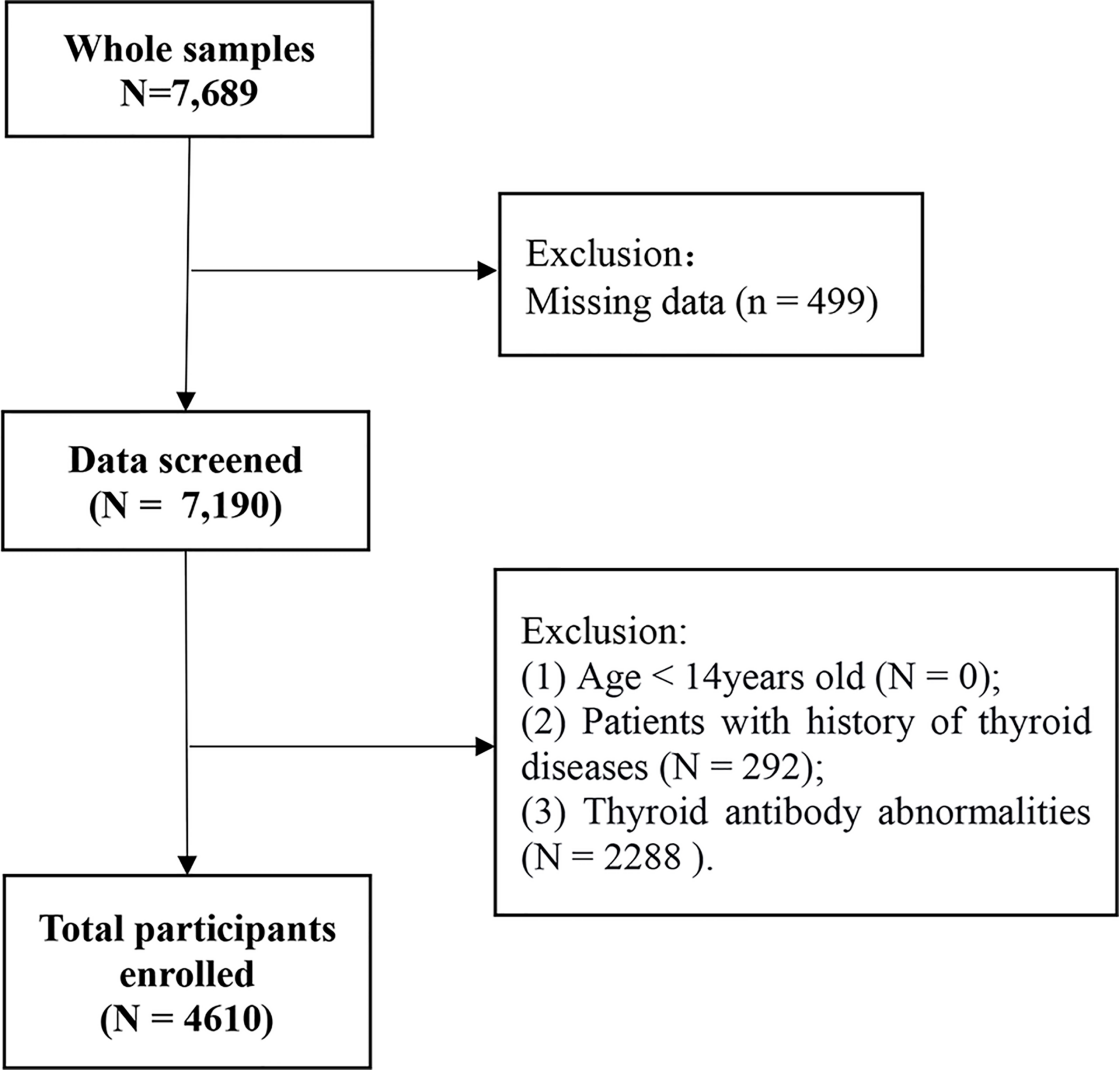
Flowchart of the inclusion and exclusion of participants.

The participants consisted of 7,689 adults (age ≥ 14 years old) who completed health examinations at the health medical center of the First Hospital of China Medical University from January 1, 2017, to December 31, 2018. Exclusion criteria: 1) Age < 14 years old (n = 0); 2) Missing data (n = 499); 3) Patients with history of thyroid diseases (n = 292); 4) Thyroid antibody abnormalities (n = 2288). After exclusion, 4,610 participants were included in the final retrospective cross-sectional analysis (**Figure 1**). The study was approved by the Ethics Committee of the First Hospital of China Medical University. An informed consent waiver was obtained for using de-identified data.

### Data Collection

The participants were examined after overnight fasting for 8-12h in the morning. 1) Gender, age (years), weight (kg), height (meter), waist circumference (WC), systolic blood pressure (SBP, mmHg), and previous medical history of the participants were measured and recorded. 2) Body mass index (BMI) is derived by dividing the weight in kilograms by the squared height in meters (kg/m^2^). 3) WC was determined at mid-abdomen (midpoint between subcostal and suprailiac landmarks according to WHO protocol) (19). 4) BP was measured after at least five minutes of rest and averaged twice BP reading measured at an interval of two minutes.

### Biochemical Measurements

An automatic biochemical analyzer (Hitachi, Japan) was utilized for biochemical parameters measurement, including fasting plasma glucose (FPG), triglyceride (TG), total cholesterol (TC), high-density lipoprotein cholesterol (HDL-C), and low-density lipoprotein cholesterol (LDL-C) levels. Hyper-triglyceridemia (hyper-TG), hyper-cholesterolemia (hyper-TC), hypo-high-density lipoprotein cholesterolemia (hypo-HDL), hyper-low-density lipoprotein cholesterolemia (hyper-LDL) were defined as TG ≥ 1.7 mmol/L, TC ≥ 5.2 mmol/L, HDL-C < 1.0 mmol/L, and LDL-C ≥ 3.4 mmol/L, respectively, and dyslipidemia if any one of them (20). Serum levels of FT_3_, FT_4_, TSH, thyroid peroxidase antibody (TPOAb), and thyroglobulin antibody (TgAb) were determined by electrochemiluminescent immunoassays on Architect i2000SR (Abbott Laboratories, Chicago, IL, USA). The reference ranges of FT_3_, FT_4_, and TSH were 2.63-5.70 pmol/L, 9.01-19.05 pmol/L, and 0.35-4.94 mU/L, respectively. The thyroid antibody abnormality was defined as TPOAb ≥ 5.61 IU/ml and/or TGAb ≥ 4.11 IU/ml. TFQI_FT4_ is achieved by the algorithm TFQI = cumulative distribution function (cdfFT_4_) – (1 – cdfTSH) (18). In order to investigate the role of FT_3_ in this index, FT_4_ in TFQI_FT4_ formulas was replaced with FT_3_ to obtain TFQI_FT3_. The value of TFQI ranged from -1 to 1. For TFQI, negative values indicated that the hypothalamus-pituitary-thyroid axis was more sensitive to the change of thyroid hormones; positive values indicated low sensitivity; the value of 0 indicated a normal sensitivity.

### Abdominal Ultrasonography and NAFLD Definition

Abdominal ultrasonography (USG) was used to test liver disease. All participants underwent abdominal USG (Siemens Acuson X300, German). NAFLD was defined by at least two of the following positive ultrasound finding (1. The liver near-field echogenicity is enhanced diffusely and is stronger than that of the kidney; 2. The structure of the intrahepatic duct is blurring; 3. The liver far-field echogenicity weakened gradually), and no history of heavy drinking (weekly alcohol intake ≤ 210g in males and ≤ 140g in females) (21).

### Statistical Analysis

The data were processed using SPSS 22.0 statistical software. Continuous variables with normal distribution were shown as means ± standard deviation (SD), and the independent T-test was performed to compare groups. While continuous variables with skewed distribution were shown as medians (interquartile ranges), non-parametric Mann-Whitney tests were conducted to compare groups. All categorical variables were expressed as relative numbers, and the χ2 tests were used to compare groups. Kendall's tau-b was used to calculate the correlation coefficient. Correlation is generally defined as very weak if correlation coefficient (r) <0.2, weak if r ≥0.2 and <0.4, moderate if r ≥0.4 and <0.6, strong if r ≥0.6 and <0.8, and very strong if r ≥0.8. To evaluate the association between thyroid parameters with lipid profiles and NAFLD, logistic regression models were used. Model 1 adjusted for demographic factors, including gender and age; model 2 adjusted all the factors adjusted in model 1 plus metabolic factors, including BMI, WC, SBP, and FPG. To evaluate the performance of the indices, we examined the receiver operating characteristics curves (ROC), which plots sensitivity against 1-specificity, and calculated the cut-points from ROC results. All calculated *P* values were two-sided, and a *P* value < 0.05 was taken to indicate a significant difference.

## Results

### Clinical Characteristics of the Participants

The clinical baseline data of participants are shown in detail in **Table 1**. A total of 4,610 participants were included in the final analysis, 2681 men (58.2%) with an average age of 47.88 ± 11.19 years. The incidence of dyslipidemia was 62.4%, higher in men than in women (66.9% *vs.* 33.1%, *P*< 0.001). Compared with the normal lipid profiles group, the age, BMI, WC, SBP, FPG, FT_3_, FT_3_/FT_4_, TFQI_FT3_, TG, TC, and LDL-C levels in the dyslipidemia group were significantly higher (*P* < 0.01), the FT_4_ and HDL-C levels in the dyslipidemia group were significantly lower (*P* < 0.01). The difference of TSH and TFQI_FT4_ between the two groups was not statistically significant (*P* = 0.568, *P* = 0.130, respectively).

**Table 1:** Comparison of clinical characteristics between participants with and without dyslipidemia.

	All	Dyslipidemia group	Normal lipid profiles group	*P*
N (%)	4610 (100)	2877 (62.4)	1733 (37.6)	–
Gender (Men/Women)	2681/1929	1926/951	755/978	**<0.001**
Age (years )	47.88±11.19	49.35±10.47	45.44±11.89	**<0.001**
BMI (Kg/m^2^)	25.23±3.52	26.05±3.35	23.87±3.39	**<0.001**
WC (cm)	83.97±10.77	86.73±10.12	79.38±10.25	**<0.001**
SBP (mmHg)	128.84±19.06	131.74±18.62	124.01±18.81	**<0.001**
FPG (mmol/L)	5.16 (4.83,5.59)	5.25 (4.91,5.74)	5.02 (4.73,5.38)	**<0.001**
FT_3_ (pmol/L)	4.39±0.54	4.42±0.54	4.33±0.54	**<0.001**
FT_4_ (pmol/L)	13.35±1.55	13.31±1.54	13.44±1.58	**0.006**
FT_3_/FT_4_	0.33±0.05	0.34±0.05	0.32±0.04	**<0.001**
TSH (mIU/L)	1.56 (1.12,2.17)	1.56 (1.11,2.17)	1.54 (1.12,2.16)	0.568
TFQI_FT3_	0±0.39	0.02±0.39	-0.04±0.39	**<0.001**
TFQI_FT4_	0±0.38	-0.01±0.38	0.01±0.38	0.130
TPOAb (IU/ml)	0.29 (0.12,0.58)	0.27 (0.11,0.57)	0.31 (0.14,0.61)	**0.006**
TGAb (IU/ml)	1.46 (1.04,2.03)	1.42 (1.02,1.98)	1.53 (1.09,2.12)	**<0.001**
TG (mmol/L)	1.34 (0.89,2.09)	1.87 (1.26,2.59)	0.90 (0.66,1.21)	**<0.001**
TC (mmol/L)	4.94±0.90	5.29±0.91	4.37±0.50	**<0.001**
HDL-C (mmol/L)	1.32±0.37	1.23±0.38	1.46±0.32	**<0.001**
LDL-C (mmol/L)	3.10±0.80	3.41±0.79	2.58±0.48	**<0.001**

Data are means ± standard deviations or medians (interquartile ranges) for continuous variables, and numbers (proportions) for categorical variables. P values are calculated by t-test and Mann–Whitney tests for continuous variables, Chi-square test for categorical variables.

BMI, body mass index; WC, waist circumference; SBP, systolic blood pressure; FPG, fasting plasma glucose; FT_3_, free triiodothyronine; FT_4_, free thyroxine; FT_3_/FT_4_, FT_3_ to FT_4_ ratio; TSH, thyroid stimulating hormone; TFQI_FT3_, the thyroid feedback quantile-based index calculated by FT_3_; TFQI_FT4_, the thyroid feedback quantile-based index calculated by FT_4_; TPOAb, thyroid peroxidase antibody; TgAb, thyroglobulin antibody; TG, triglyceride; TC, total cholesterol; HDL-C, high-density lipoprotein-cholesterol; LDL-C, low-density lipoprotein-cholesterol.

Bold values emphasized that P<0.05.

**Table 2** showed that NAFLD incidence in the participants was 48.9%, higher in men than in women (75.5% *vs.* 24.5%, *P* < 0.001). Compared with the control group, the age, BMI, WC, SBP, FPG, FT_3_, FT_3_/FT_4_, TFQI_FT3_, TG, TC, and LDL-C levels in the NAFLD group were significantly higher, while the HDL-C levels in the NAFLD group were significantly lower (*P* < 0.01). There was no significant difference in FT_4_, TSH, and TFQI_FT4_ levels between the NAFLD group and the control group (*P* = 0.078, *P* = 0.320, *P* = 0.091, respectively).

**Table 2:** Comparison of clinical characteristics between participants with and without NAFLD.

	NAFLD group	Control group	*P*
N(%)	2252 (48.9)	2358 (51.1)	–
Gender (Men/Women)	1701/551	980/1378	**<0.001**
Age (years )	48.87±10.23	46.93±11.95	**<0.001**
BMI (Kg/m^2^)	27.09±3.07	23.45±2.97	**<0.001**
WC (cm)	89.43±9.02	78.75±9.67	**<0.001**
SBP (mmHg)	133.67±17.99	124.22±18.92	**<0.001**
FPG (mmol/L)	5.34 (4.99,5.91)	5.00 (4.71,5.36)	**<0.001**
FT3 (pmol/L)	4.46±0.51	4.32±0.56	**<0.001**
FT4 (pmol/L)	13.31±1.53	13.39±1.58	0.078
FT_3_/FT_4_	0.34±0.05	0.33±0.05	**<0.001**
TSH (mIU/L)	1.55 (1.12,2.13)	1.56 (1.11,2.21)	0.320
TFQI_FT3_	0.04±0.39	-0.04±0.39	**<0.001**
TFQI_FT4_	0±0.38	0±0.38	0.091
TPOAb (IU/ml)	0.29 (0.11,0.58)	0.29 (0.12,0.58)	0.629
TGAb (IU/ml)	1.42 (1.01,1.98)	1.50 (1.06,2.07)	**0.001**
TG (mmol/L)	1.88 (1.30,2.67)	1.01 (0.72,1.44)	**<0.001**
TC (mmol/L)	5.07±0.92	4.82±0.87	**<0.001**
HDL-C (mmol/L)	1.17±0.31	1.46±0.37	**<0.001**
LDL-C (mmol/L)	3.24±0.81	2.96±0.77	**<0.001**

Data are means ± standard deviations or medians (interquartile ranges) for continuous variables, and numbers (proportions) for categorical variables. P values are calculated by t-test and Mann–Whitney tests for continuous variables, Chi-square test for categorical variables.

BMI, body mass index; WC, waist circumference; SBP, systolic blood pressure; FPG, fasting plasma glucose; FT_3_, free triiodothyronine; FT_4_, free thyroxine; FT_3_/FT_4_, FT_3_ to FT_4_ ratio; TSH, thyroid stimulating hormone; TFQI_FT3_, the thyroid feedback quantile-based index calculated by FT_3_; TFQI_FT4_, the thyroid feedback quantile-based index calculated by FT_4_; TPOAb, thyroid peroxidase antibody; TgAb, thyroglobulin antibody; TG, triglyceride; TC, total cholesterol; HDL-C, high-density lipoprotein-cholesterol; LDL-C, low-density lipoprotein-cholesterol.

Bold values emphasized that P<0.05.

### Correlation Between Thyroid Parameters and Lipid Profiles

FT_3_ levels were positively correlated with TG, TC, and LDL-C levels and negatively correlated with HDL-C levels (r = 0.109, *P* < 0.001, r = 0.031, *P* = 0.002, r = 0.048, *P* < 0.001, r = -0.084, *P* < 0.001, respectively). However, FT_4_ levels were negatively correlated with TG levels and positively correlated with HDL-C levels (r = -0.044, *P* < 0.001 and r = 0.023, *P* = 0.022, respectively). While TSH levels were positively correlated with TG and TC levels (r = 0.020, *P* = 0.040, r = 0.025, *P* = 0.012) (**Table 3**). FT_3_/FT_4_ was positively correlated with TG, TC, and LDL-C levels (r = 0.120, *P* < 0.001, r = 0.029, *P* = 0.003, r = 0.043, *P* < 0.001, respectively) and negatively correlated with HDL-C levels (r = -0.089, *P* < 0.001), results from TFQI_FT3_ were similar with FT_3_/FT_4_. In contrast, TFQI_FT4_ was only positively correlated with HDL-C levels (r = 0.03, *P* = 0.003).

**Table 3:** Correlation between thyroid parameters and lipid profiles.

		FT_3_	FT_4_	TSH	FT_3_/FT_4_	TFQI_FT3_	TFQI_FT4_
TG	r	0.109	-0.044	0.020	0.120	0.095	-0.016
	*P*	**<0.001**	**<0.001**	**0.040**	**<0.001**	**<0.001**	0.100
TC	r	0.031	-0.004	0.025	0.029	0.040	0.016
	*P*	**0.002**	0.711	**0.012**	**0.003**	**<0.001**	0.096
HDL-C	r	-0.084	0.023	0.018	-0.089	-0.049	0.030
	*P*	**<0.001**	**0.022**	0.063	**<0.001**	**<0.001**	**0.003**
LDL-C	r	0.048	-0.003	0.013	0.043	0.044	0.009
	*P*	**<0.001**	0.769	0.180	**<0.001**	**<0.001**	0.362

Kendall's tau-b was used to calculate the correlation coefficient (r).

TG, triglyceride; TC, total cholesterol; HDL-C, high-density lipoprotein-cholesterol; LDL-C, low-density lipoprotein-cholesterol. FT_3_, free triiodothyronine; FT_4_, free thyroxine; TSH, thyroid stimulating hormone; FT_3_/FT_4_, FT_3_ to FT_4_ ratio; TFQI_FT3_, the thyroid feedback quantile-based index calculated by FT_3_; TFQI_FT4_, the thyroid feedback quantile-based index calculated by FT_4_.

Bold values emphasized that P<0.05.

### Association of Thyroid Parameters With Dyslipidemia and NAFLD

We performed gender and age-adjusted and multivariate-adjusted models with the inclusion of thyroid function parameters and sensitivity to thyroid hormone indices (**Table 4**). After adjustment for gender and age in model 1, we found that the FT_3_, FT_3_/FT_4_, and TFQI_FT3_ were positively associated with the risks of hyper-TG, hyper-TC, hyper-LDL, and NAFLD (*P* < 0.05). The TSH was also positively associated with risks of hyper-TG, hyper-TC, and hyper-LDL (*P* < 0.05), however, TSH was not significantly associated with the risk of NAFLD (*P* = 0.05). Moreover, FT_4_ showed a negative association with risks of hyper-TG and NAFLD (*P* < 0.05). Furthermore, all the results remained in model 2 except for the association between FT_4_ with the risk of NAFLD (*P* =0.163).

**Table 4:** Logistic regression analysis of the association between thyroid parameters with dyslipidemia and NAFLD.

	Thyroid parameters	n/N	Model 1		Model 2	
			OR(95% CI)	*P*	95% CI	*P*
Hyper-TG						
	FT_3_	1696/4610	1.24 (1.10, 1.40)	**0.001**	1.23 (1.08, 1.40)	**0.002**
	FT_4_	1696/4610	0.90 (0.87, 0.94)	**<0.001**	0.91 (0.87, 0.95)	**<0.001**
	TSH	1696/4610	1.12 (1.05, 1.19)	**<0.001**	1.09 (1.02, 1.16)	**0.009**
	FT_3_/FT_4_			**<0.001**		**<0.001**
	Q1	447/1538	1		1	
	Q2	552/1537	1.28 (1.10, 1.50)	**0.002**	1.34 (1.14, 1.59)	**<0.001**
	Q3	697/1535	1.66 (1.42, 1.94)	**<0.001**	1.64 (1.40, 1.94)	**<0.001**
	TFQI_FT3_	1696/4610	1.55 (1.32, 1.83)	**<0.001**	1.41 (1.19, 1.68)	**<0.001**
	TFQI_FT4_	1696/4610	0.92 (0.78, 1.09)	0.336	0.84 (0.71, 1.00)	0.055
Hyper-TC						
	FT_3_	1730/4610	1.24 (1.10, 1.40)	**<0.001**	1.23 (1.09, 1.38)	**0.001**
	FT_4_	1730/4610	1.00 (0.96, 1.04)	0.990	1.00 (0.96, 1.04)	0.886
	TSH	1730/4610	1.10 (1.04, 1.16)	**0.002**	1.08 (1.02, 1.15)	**0.007**
	FT_3_/FT_4_			**0.003**		**0.005**
	Q1	528/1538	1		1	
	Q2	594/1537	1.22 (1.05, 1.41)	**0.010**	1.22 (1.05, 1.42)	**0.008**
	Q3	608/1535	1.29 (1.11, 1.49)	**0.001**	1.26 (1.09, 1.47)	**0.003**
	TFQI_FT3_	1730/4610	1.36 (1.17, 1.59)	**<0.001**	1.30 (1.11, 1.52)	**0.001**
	TFQI_FT4_	1730/4610	1.15 (0.98, 1.34)	0.095	1.11 (0.94, 1.30)	0.209
Hypo-HDL						
	FT_3_	844/4610	0.89 (0.76, 1.04)	0.133	0.90 (0.77, 1.05)	0.183
	FT_4_	844/4610	0.95 (0.91, 1.00)	0.053	0.96 (0.91, 1.01)	0.107
	TSH	844/4610	1.00 (0.92, 1.08)	0.987	0.98 (0.90, 1.07)	0.665
	FT_3_/FT_4_			0.584		0.427
	Q1	237/1538	1		1	
	Q2	277/1537	1.08 (0.89, 1.32)	0.439	1.14 (0.93, 1.40)	0.199
	Q3	330/1535	1.10 (0.91, 1.34)	0.316	1.10 (0.90, 1.34)	0.362
	TFQI_FT3_	844/4610	0.96 (0.79, 1.18)	0.703	0.93 (0.75, 1.14)	0.484
	TFQI_FT4_	844/4610	0.92 (0.75, 1.13)	0.420	0.89 (0.72, 1.10)	0.284
Hyper-LDL						
	FT_3_	1576/4610	1.17 (1.04, 1.32)	**0.009**	1.15 (1.02, 1.30)	**0.021**
	FT_4_	1576/4610	1.00 (0.96, 1.04)	0.861	1.00 (0.97, 1.05)	0.828
	TSH	1576/4610	1.10 (1.03, 1.16)	**0.002**	1.08 (1.02, 1.14)	**0.015**
	FT_3_/FT_4_			**0.011**		**0.028**
	Q1	475/1538	1		1	
	Q2	543/1537	1.21 (1.04, 1.41)	**0.013**	1.21 (1.04, 1.41)	**0.015**
	Q3	558/1535	1.24 (1.06, 1.45)	**0.006**	1.20 (1.02, 1.40)	**0.025**
	TFQI_FT3_	1576/4610	1.28 (1.09, 1.50)	**0.003**	1.19 (1.01, 1.40)	**0.034**
	TFQI_FT4_	1576/4610	1.10 (0.93, 1.29)	0.257	1.02 (0.86, 1.21)	0.812
NAFLD						
	FT_3_	2252/4610	1.25 (1.10, 1.41)	**<0.001**	1.24 (1.08, 1.44)	**0.003**
	FT_4_	2252/4610	0.94 (0.91, 0.98)	**0.004**	0.97 (0.93, 1.01)	0.163
	TSH	2252/4610	1.06 (1.00, 1.13)	0.050	1.00 (0.93, 1.07)	0.923
	FT_3_/FT_4_			**<0.001**		**<0.001**
	Q1	633/1538	1		1	
	Q2	742/1537	1.24 (1.06, 1.44)	**0.006**	1.32 (1.11, 1.57)	**0.002**
	Q3	877/1535	1.49 (1.28, 1.74)	**<0.001**	1.39 (1.17, 1.66)	**<0.001**
	TFQI_FT3_	2252/4610	1.48 (1.26, 1.73)	**<0.001**	1.24 (1.03, 1.49)	**0.024**
	TFQI_FT4_	2252/4610	1.01 (0.86, 1.19)	0.926	0.90 (0.75, 1.09)	0.274

Model 1 adjusted for demographic factors, including gender and age; model 2 adjusted all the factors adjusted in model 1 plus metabolic factors, including BMI, WC, SBP, and FPG. CI, confidence interval; FT_3_, free triiodothyronine; FT_4_, free thyroxine; TSH, thyroid stimulating hormone; FT_3_/FT_4_, FT_3_ to FT_4_ ratio (Q1, 0< FT3/FT4 ≤ 0.31; Q2, 0.31< FT3/FT4 ≤ 0.35; Q3, FT3/FT4>0.35); TFQI_FT3_, the thyroid feedback quantile-based index calculated by FT_3_; TFQI_FT4_, the thyroid feedback quantile-based index calculated by FT_4_; Hyper-TG, hyper-triglyceridemia; Hyper-TC, hyper-cholesterolemia; Hypo-HDL, hypo-high-density lipoprotein cholesterolemia; Hyper-LDL, hyper-low high-density lipoprotein cholesterolemia.

Bold values emphasized that P<0.05.

### ROC Curves for Optimal Cut-Points of TFQI_FT3_ and FT_3_/FT_4_

**Figure 2:**
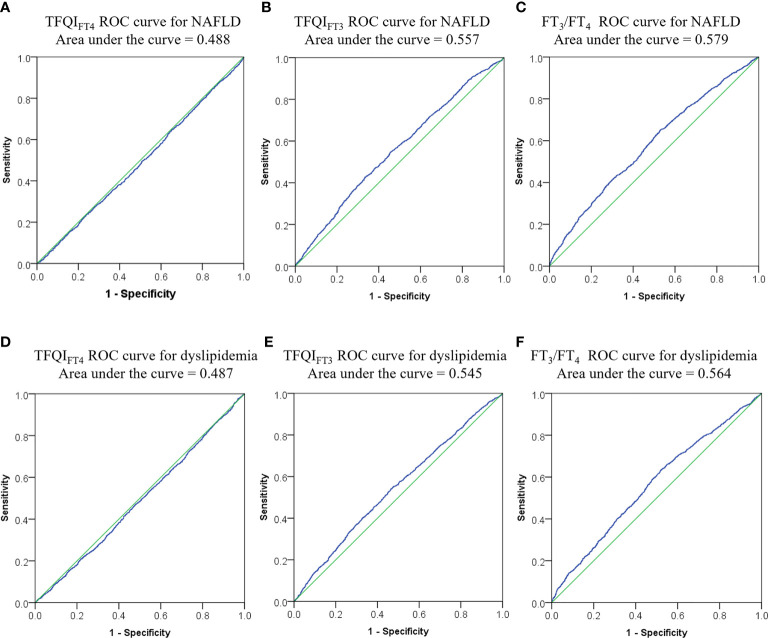
ROC curves for optimal cut-points of TFQI_FT3_, and FT_3_/FT_4_. **(A–C)** ROC curve for NAFLD from TFQI_FT4_, TFQI_FT3_, and FT_3_/FT_4_; **(D–F)** ROC curve for dyslipidemia from TFQI_FT4_, TFQI_FT3_, and FT_3_/FT_4_. ROC, receiver operating characteristic, FT_3_, free triiodothyronine; FT_4_, free thyroxine; FT_3_/FT_4_, FT_3_ to FT_4_ ratio; TFQI_FT3_, the thyroid feedback quantile-based index calculated by FT_3_; TFQI_FT4_, the thyroid feedback quantile-based index calculated by FT_4_.

**Figure 2** showed that TFQI_FT3_ and FT_3_/FT_4_ performed better than TFQI_FT4_ on ROC analyses for NAFLD prediction (area under ROC curve 0.557, P < 0.001; 0.579, P < 0.001; 0.488, P = 0.149 respectively). The optimal cut-points of TFQI_FT3_ and FT_3_/FT_4_ for NAFLD prediction were 0.120 and 0.319. However, both TFQI_FT3_ and FT_3_/FT_4_ yielded very low diagnostic sensitivity and specificity for NAFLD prediction at the optimal cut-points (0.43, 0.66; 0.65, 0.47, respectively). Although TFQI_FT3_ and FT_3_/FT_4_ also performed better than TFQI_FT4_ on ROC analyses for dyslipidemia prediction, the area under ROC curve is relatively small (area under ROC curve 0.545, P < 0.001; 0.564, P < 0.001; 0.487, P = 0.131 respectively).

## Discussion

As far as we know, this is the first study to evaluate the association between central and peripheral sensitivity to thyroid hormone indices with the risk of NAFLD. This cross-sectional study demonstrated that FT_3_/FT_4_ and TFQI_FT3_ levels were positively associated with the risk of hyper-TG, hyper-TC, hyper-LDL, and NAFLD. In contrast, no association was observed between TFQI_FT4_ with the risks of dyslipidemia and NAFLD. The novelty of the present study is to apply the sensitivity of thyroid hormones indices rather than absolute circulating values of FT_3_, FT_4_, and TSH as a predictor of NAFLD risk, which could be more informative, directly correlating thyroid hormone resistance to hepatic metabolic alteration, giving advancement in current knowledge. Moreover, we introduced the TFQI_FT3_ index by substituting FT_3_ from the calculation of TFQI_FT4_. Such TFQI_FT3_ index along with FT_3_/FT_4_ is closely related to the risk of dyslipidemia and NAFLD.

Numerous previous studies have found that thyroid dysfunction, including hypothyroidism and hyperthyroidism, is significantly associated with dyslipidemia (22). Elevated plasma TC, LDL-C, TG level, and decreased plasma HDL-C level can be found in hypothyroidism. The plasma levels of TC and LDL-C show the most pronounced changes (23–25). In comparison, the opposite blood lipid level can be found in hyperthyroidism.

Thyroid hormones affect lipid metabolism manifold, such as synthesis, mobilization, and degradation (25). Thyroid hormones can stimulate 3-hydroxy-3methylglutarylcoenzyme A reductase, which initiates cholesterol biosynthesis (26). Additionally, triiodothyronine (T3) can bind to specific thyroid hormone-responsive elements to activate the LDL receptor gene, thus upregulates LDL receptors (27). Moreover, Thyroid hormones can regulate cholesterol metabolism by increasing the expression of the regulatory sterol element-binding protein-2 (SREBP-2) (28). Furthermore, Thyroid hormones can also regulate HDL metabolism; previous studies revealed that thyroid hormones exchanged cholesteryl esters from HDL2 to the very low-density lipoproteins and TGs in the opposite direction by increasing cholesteryl ester transfer activity (26). Another effect of T3 is to stimulate lipoprotein lipase, which catabolizes the TG-rich lipoproteins, leading to a decrease of TG (26).

It is biologically plausible that thyroid hormones exert significant effects on the development of NAFLD. As we mentioned above, thyroid hormones had multiple effects on lipid metabolism at both systemic and hepatic levels by virtue of their roles in regulating the circulating level of lipoprotein, TG, and TC, as well as hepatic TG accumulation and metabolism (7, 29). Recent studies showed that the expression of hepatic lipogenic genes was regulated by thyroid hormones, what is more, several genes whose expression is changed in NAFLD were also regulated by thyroid hormones (30, 31). Additionally, decreased hepatic levels of thyroid hormones and defective intrahepatic deiodinase expression were found in NAFLD (32). On the other side, the previous study showed that excessive hepatic fatty acids in NAFLD may damage the activity of thyroid hormone receptors (33). Moreover, this apparent local hypothyroid status promotes hepatic triglyceride accumulation by decreasing hepatic lipase activity (34). Furthermore, *in vivo* studies have shown that not only thyroid hormone administration but also thyroid hormone agonists ameliorates hepatic fat accumulation (35–37).

TSH level is one of the essential risk factors in the pathogenesis of NAFLD, independent of FT_3_ and FT_4_. Tahara et al. found that the serum TSH level was significantly associated with the risk of NAFLD, while FT_4_ was not significantly related to the risk of NAFLD in the subclinical hypothyroidism population (7). Chung et al. found that NAFLD was positively associated with TSH serum level. They revealed that subclinical hypothyroidism was closely associated with the risk of NAFLD in a TSH dose-dependent manner, even within the normal upper TSH level range (38). Liu et al. showed that the serum levels of TSH in patients with NASH or without NASH were different significantly. Furthermore, the NASH prevalence in patients with subclinical hypothyroidism was significantly higher than in the euthyroidism patients. In multivariate analyses, they concluded that elevated serum TSH levels predicted the risk of NASH independently (39). Additionally, Kim et al. reported that even within the normal range of T4, an increase in the TSH level was closely related to the biopsy-proven NASH and advanced fibrosis (40).

As we mentioned above, positive associations of FT3 and TSH level with the risk of NAFLD, negative associations of FT_4_ level with the risk of NAFLD suggest that the role of thyroid hormone in the development and progression of NAFLD is complex. This association is at odds with the physiological effects of thyroid hormones, which are considered capable of activating lipolysis. Thus, we speculate that the contradictory results may reflect the close association between sensitivity to thyroid hormone with NAFLD.

TSH, FT_4_, and FT_3_ are closely regulated and influenced by each other. Compared with a single index, the calculation of composite indices can systematically reflect the regulation of thyroid hormone homeostasis. Our results showed that FT_3_/FT_4_ was significantly positively associated with the risks of hyper-TG, hyper-TC, hyper-LDL, and NAFLD. FT_4_ can be converted to FT_3_ by deiodinase in the peripheral. Thus FT_3_/FT_4_ can be considered as an indicator of peripheral deiodinase activity. A previous study by Bilgin and Pirgon suggested that the augmented conversion from FT_4_ to FT_3_ by increasing deiodinase activity was a compensatory mechanism for fat excessively accumulation to ameliorate energy expenditure (41). Consistent with our study, Gokmen et al. found that the patients with NAFLD had significantly elevated FT_3_/FT_4_, and FT_3_/FT_4_ is a independent predictor of NAFLD in euthyroid patients and hyperthyroid patients (42).

In 2019, Laclaustra et al. proposed a new sensitivity to thyroid hormone index (TFQI) to detect mild levels of acquired thyroid hormone resistance in the population; the result showed that TFQI was more stable than the TSH index and TSH T4 index in evaluating sensitivity to thyroid hormone. That study also showed that TFQI values were related to obesity, diabetes, metabolic syndrome, and diabetes-related mortality (18). As we know, the prevalence of NAFLD is related to multiple metabolic risk factors, such as obesity, diabetes, and so on (4). NAFLD is also a strong determinant for the development of metabolic syndrome (43); what is more, metabolic abnormalities in metabolic syndrome, including diabetes, obesity, and hyperlipidemia, are critical metabolic risk factors for NAFLD (44, 45). Thus, in the present study, we proposed that TFQI might be related to NAFLD and might be a diagnostic predictor for NAFLD. In our study, sensitivity to thyroid hormone evaluation by the TFQI_FT3_ was significantly positively associated with the risk of hyper-TG, hyper-TC, hyper-LDL, and NAFLD. Moreover, TFQI_FT3_ and FT_3_/FT_4_ performed better than TFQI_FT4_ on ROC analysis; although, TFQI_FT3_ and FT_3_/FT_4_ yielded low diagnostic sensitivity and specificity. In comparison, no association was found between TFQI_FT4_ with the risk of dyslipidemia and NAFLD. Although the exact mechanisms remain unclear, the following aspect might be the possible explanation: serum level of FT3, which is mainly conversed from serum FT_4_ by deiodinase, can be considered as a compensatory mechanism for fat accumulation to improve energy expenditure and reflect better sensitivity of thyroid hormone (41). Thyroid function is also race-specific; in the present study, we only included Chinese participants. Therefore, the contradictory results in our study may be partly due to interethnic variations.

There are still some limitations in the present study: 1) The present study was designed cross-sectionally. Thus we only found the association between sensitivity to thyroid hormone indices with risk of NAFLD, and the design limited our ability to collect the follow-up data and evaluate the causality of associations;2) Liver biopsy was not used to accurately detect NAFLD, while ultrasonography was utilized to diagnosed NAFLD, there was limited accuracy for detecting mild hepatic lipid accumulation; 3) this study included only Chinese patients who completed health examinations at a single medical center. Since the limitation mentioned, the present results above still need further confirmation by longitudinal prospective studies in multiple race populations.

## Conclusions

The present study showed that TFQI_FT3_ and FT_3_/FT_4_ were independently associated with the risk of dyslipidemia and NAFLD after multiple adjustments. TFQI_FT3_ and FT_3_/FT_4_ performed better than TFQI_FT4_ on ROC analyses for dyslipidemia and NAFLD prediction. Thus TFQI_FT3_ and FT_3_/FT_4_ can be used as new indicators for predicting dyslipidemia and NAFLD, although the diagnostic sensitivity and specificity at the optimal cut-points are very low, while TFQI_FT4_ has insufficient evidence in predicting dyslipidemia and NAFLD.

## Data Availability Statement

The original contributions presented in the study are included in the article. Further inquiries can be directed to the corresponding authors.

## Ethics Statement

The study was approved by the Ethics Committee of the First Hospital of China Medical University. The ethics committee waived the requirement of written informed consent for participation.

## Author Contributions

SL, JL, and ZW conducted a literature search, assisted with study design, data collection, data analysis, data interpretation, and draft the manuscript. WW and HG participated in study design, and revised the manuscript. All authors contributed to the article and approved the submitted version.

## Conflict of Interest

The authors declare that the research was conducted in the absence of any commercial or financial relationships that could be construed as a potential conflict of interest.

## Publisher's Note

All claims expressed in this article are solely those of the authors and do not necessarily represent those of their affiliated organizations, or those of the publisher, the editors and the reviewers. Any product that may be evaluated in this article, or claim that may be made by its manufacturer, is not guaranteed or endorsed by the publisher.
